# Methodology for UK recruitment into a large-scale international clinical trial

**DOI:** 10.1186/1745-6215-16-S2-P122

**Published:** 2015-11-16

**Authors:** Elizabeth Wincott, Rejive Dayanandan, Richard Haynes, Michael Lay, Martin Landray, Jane Armitage

**Affiliations:** 1 University of Oxford, Oxford, UK

## Background

Recruitment into long-term trials requires identification and screening of potential subjects and strategies to maximize post-randomization compliance. We describe a successful strategy used for UK recruitment into a large international trial assessing the effect of extended release niacin/laropiprant (ERN/LRPT) on cardiovascular outcomes.

## Methods

Ethics and Section 251 of the Health and Social Care Act approval allowed potentially eligible patients to be identified (without consent) from local site's hospital records and invited by a central coordinating office to a local screening appointment.

Following a screening visit and initial run-in phase to standardise background LDL-cholesterol management, those remaining eligible entered an active ERN/LRPT run-in to assess their tolerance of the drug and likely long-term compliance. Participants, if still eligible, were then randomized to ERN/LRPT or placebo.

## Results

Electronic records from 89 hospitals allowed 228,391 potential participants to be invited of whom 24,396 (11%) attended a Screening visit. Of the 14,237 entering the active ERN/LRPT run-in, 6202 (44%) withdrew before randomization; the majority (82%) due to adverse effects from ERN/LRPT. Without this active run-in, randomization of the 8035 participants would have been completed in about half the time but adherence to study treatment and completeness of follow-up after randomization would have been substantially worse than the 77% and > 99% achieved respectively, adversely affecting power.

## Conclusion

This recruitment method provided a streamlined, cost-effective approach to successful large-scale recruitment. The active pre-randomization run-in ensured that only those able to tolerate the drug were randomized helping to ensure good post-randomization adherence.

